# Burnout Among the Clinical Dental Students in the Jordanian Universities

**DOI:** 10.4021/jocmr2009.09.1263

**Published:** 2009-10-16

**Authors:** Wala Majid Amin, Muna H. Al-Ali, Ramzi B. Duaibis, Tamara Oweis, Darwish H. Badran

**Affiliations:** a Faculty of Dentistry, University of Jordan.; b Department of Endodontics, Faculty of Dentistry, University of Melbourne, Australia; c Department of Orthodontics, Faculty of Dentistry, University of Illinois, USA; d Center for Educational Development, University of Jordan.

## Abstract

**Background:**

The study aimed to evaluate the level of burnout among the clinical dental students in two Jordanian universities.

**Methods:**

A total of 307 students from the two schools were surveyed using Maslach Burnout Inventory survey. Scores for the inventory’s subscales were calculated and the mean values for the students’ groups were computed separately. Kruskal-Wallis and Mann-Whitney tests were carried out and the results were compared at 95% confidence level.

**Results:**

The results showed that the dental students in both Jordanian universities suffered high levels of emotional exhaustion and depersonalization compared to reported levels for dental students in other countries. The dental students of the University of Jordan demonstrated a significantly higher (p < 0.05) level of emotional exhaustion than their counterparts in the Jordan University of Science and Technology.

**Conclusions:**

The findings indicated that dental students in the Jordanian universities presented considerable degrees of burnout manifested by high levels of emotional exhaustion and depersonalization. Studies targeting students health and psychology should be carried out to determine the causes of burnout among dental students. The curricula of the dental schools in the two universities should be accordingly improved to minimize burnout among the students.

**Keywords:**

Burnout; Emotional exhaustion; Depersonalization; Personal accomplishment; Maslach Burnout Inventory

## Introduction

Dentistry is a profession demanding physical and mental efforts as well as people contacts.

Excessive contact and handling of people can result in a condition known as Burnout. Burnout, therefore, is defined as ‘a syndrome of emotional exhaustion and cynicism that occurs frequently among individuals who do ‘people-work’ of some kind’ [1]. Burnout is characterized by three key aspects: emotional exhaustion (mental fatigue), depersonalization (psychological distancing from others), and reduced personal accomplishment [2,3].

The rate of burnout among dentists and its effect on their lives have been previously investigated by many researchers [4-12]. It has been found that burnout can lead to early retirement, indifference to treatment outcomes, and to patient condition or needs [4]. The affected dentist also tends to avoid any contact with people, whether they are colleagues, patients, friends, or even family. To this end, burnout obviously has serious detrimental effects on the profession as well as on the society [3,6].

Many researchers studied the existence of stress among dental students [2,7-12], a few studied the prevalence of burnout [2,7-9,12] but none to our knowledge studied its effect on their learning abilities and performance.

In a study aimed at testing the rate of burnout among German dental students of three universities [3], it was found that, regardless of the educational system used, there were high scores of burnout among students. In a separate study, emotional exhaustion among dental students of seven European schools was found significantly higher than the reported scores of medical students [2].

The perceived stress among dental students had been attributed to factors such as fear of failure [7], the load of academic and clinical work [8,11], unavailability, in some schools, of materials for study and clinical training [10], performance pressure, and self-efficacy beliefs [12]. It has been reported that students who demonstrated high levels of stress tended to show lower grades for clinical competency and contextual understanding [9].

The aim of this study was to determine the presence and level of burnout among the clinical dental students in the University of Jordan and in the Jordan University of Science and Technology, and also to compare the results with those of other countries.

## Materials and Methods

The extent of burnout in 307 clinical dental students of the University of Jordan (UJ) and Jordan University of Science and Technology (JUST) was assessed by employing the Maslach Burnout Inventory - Human Services Survey (MBI-HSS) [1]. The inventory comprised 22 items (phrases) measuring three subscales of burnout: Emotional Exhaustion - EE (9 items), Depersonalization - DP (5 items), and Personal Accomplishment - PA (8 items). The participants recorded their responses by choosing a number on a scale from one to four which indicated how often they encountered what was described in the item (1: never, rarely or few times in a year; 2: once or few times in a month; 3: once or few times in a week; 4: daily). A high degree of burnout was obviously reflected by high scores on the EE and DP subscales and low scores on the PA subscale and vice versa. The three subscales of the inventory, though related, were independent and therefore the degree of burnout was expressed by the three scores of its three subscales rather than by a total score.

The employed inventory is an effective tool of proven reliability and validity in detecting the presence and assessing the degree of burnout in human services workers.The English version of the inventory was used since English is the teaching language in the two Jordanian dental schools.

The clinical students (fourth and fifth year) of both dental schools of UJ (83, 4th year and 93, 5th year) and JUST (64, 4th year and 67, 5th year) were asked to complete the inventory during one lecture at each of the universities during the second semester after the midterm examinations. The instructions were explained thoroughly to the students by the researchers. The response was totally anonymous to ensure confidentiality.

The total score for each of the three subscales was calculated for each student by calculating the sum of responses to the items in the subscale. Mean values were then calculated for each student group separately. Shapiro-Wilk test of normality indicated that the data was not normally distributed in many instances. Consequently, non-parametric statistical tests were used in comparing the results of the four student groups. Kruskal-Wallis one-way analysis of variance (ANOVA) based on ranks was used in determining whether any significant differences existed among the student groups. Multiple comparisons among the different groups were performed using Mann-Whitney rank sum test. The various data sets were rigorously treated statistically at 95% level of confidence. Statistical analysis was carried out using the Statistical Package for Social Sciences (Version 14.0, SPSS Inc., Chicago, Illinois, USA).

## Results

**Figure 1:**
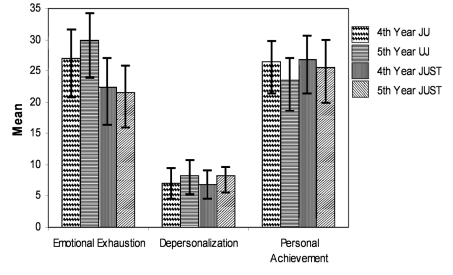
Mean scores for all students' groups for each subscale.

The responses for the 4th and 5th year students of each university were described in terms of means, standard deviations, variances, minimum and maximum values (Table 1) and (Fig. 1).

**Table 1:** Emotional exhaustion, depersonalization and personal achievement scores for all student groups

Student Group	Gender	Personal Achievement	Depersonalization	Emotional Exhaustion
		Mean ± (s.d)	Mean ± (s.d)	Mean ± (s.d)
				
4th Year UJ	Males	23.2*± (4.6)	7.0 ± (2.3)	26.7 ± (2.9)
	Females	27.3*± (5.2)	7.0 ± (2.6)	25.2 ± (4.6)
	All	26.1± (5.4)	7.0 ± (2.5)	25.6 ± (4.1)
				
5th Year UJ	Males	26.7*± (5.5)	8.1 ± (2.9)	23.6 ± (4.6)
	Females	29.9*± (4.8)	8.2 ± (2.6)	22.6 ± (4.0)
	All	29.0± (5.2)	8.1 ± (2.7)	22.9 ± (4.2)
				
4th Year JUST	Males	20.8± (5.4)	6.4 ± (2.2)	26.1 ± (4.2)
	Females	22.7± (5.2)	7.2 ± (2.3)	26.5 ± (4.0)
	All	21.8± (5.4)	6.9 ± (2.3)	26.0 ± (4.6)
5th Year JUST	Males	20.8± (4.8)	7.5 ± (2.3)	24.9 ± (4.6)
	Females	22.7± (4.9)	7.3 ± (2.3)	26.3 ± (4.0)
	All	21.0± (4.9)	7.6 ± (2.1)	24.9 ± (5.0)

* Significant difference found between males and females of the same student group at P < 0.05.

Cut-off values above which a subscale score was deemed high were obtained by correcting the values indicated in the MBI-Scoring Key to compensate for the difference in scoring scale.

Female students in UJ had higher degrees of emotional exhaustion (p < 0.05) than their male counterparts while no significant differences were found between males and females of both universities neither in depersonalization nor in personal achievement scores (Table 1).

Pairwise comparisons have shown that the 4th year UJ students exhibited significantly higher degrees of burnout (p < 0.05) in the three subscales than their 5th year counterparts. On the other hand, no significant differences were found between 4th and 5th year students in JUST. Comparisons between universities have shown that UJ students had significantly higher degrees of emotional exhaustion (p < 0.05) than their counterparts in JUST (Table 2).

**Table 2:** P values as determined by Mann-Whitney rank and sum test among the student groups for all subscales

Compared Groups	EE	DP	PA
4th UJ - 5th UJ	0.001*	0.005*	0.000*
4th JUST – 5th JUST	0.466	0.105	0.305
4th UJ – 4th JUST	0.000*	0.914	0.422
5th UJ – 5th JUST	0.000*	0.270	0.014

*Significant differences found between student groups at P < 0.05.

## Discussion

The results showed that almost all of the students of both the 4th and 5th years in the two dental schools of the two Jordanian universities suffered high degrees of emotional exhaustion. In a previous study [2] it was reported that only 22% of the surveyed students from seven European dental schools had high degrees of emotional exhaustion. The scores of emotional exhaustion determined in this study were extremely high especially those of the UJ students. Even the relatively lower scores attained by the JUST students were still higher than those reported by the European study [2]. The cause of the high levels of emotional exhaustion shown by the Jordanian dental students may, probably, be related to pressures of study, examinations, competition and fulfillment of the minimum clinical requirements in addition to the primary cause of direct contact with patients. Further investigations to determine the possible factors related to this problem and its possible causes are necessary and of significant importance.

Comparisons between the two dental faculties of the two Jordanian universities revealed that UJ students had significantly higher scores of emotional exhaustion than JUST students. Although the clinical training scheme was similar in both universities, there were some differences which were possibly in favour of JUST students. The dental faculties of both universities adopted a clinical training system which obliged the students to successfully finish a minimum amount of clinical requirements in order to pass any clinical course. Nevertheless, the minimum clinical requirements of UJ dental faculty were more in terms of quantity and variety of clinical tasks than those of JUST for 4th or 5th year students. This accounted for more physical and mental efforts of the UJ students as opposed to that of their counterparts in JUST. In addition, UJ students had to find their own patients due to the absence of patients records filing system. The dental faculty of JUST, on the other hand, organized the students patient records and distributed them according to the students needs. This had increased the number of patients the UJ students had to interact with, which consequently increased the emotional exhaustion level in those students.

Clinical sessions were longer and less frequent in JUST dental faculty. As a result, UJ students were more stressed trying to finish their procedures in time. Not to mention, that more clinical sessions per week meant more patients contacts, which in turn, increased the level of emotional exhaustion.

The availability of the staff during the clinical sessions might also have played a role. In JUST, the staff to student ratio was very favourable for both the learning experience and the students mental health. As each clinical instructor handled less number of students, the students received longer time and better attention from the staff. Moreover, JUST provided an Allied Dental Sciences program, whose students assisted the clinical dental students during their sessions by putting four-handed dentistry into practice. The dental faculty at the UJ did not offer any allied dental sciences programs. This has obliged the dental students of the UJ to look for and find their own patients and to handle them entirely by themselves, unassisted by ancillary personnel.

Depersonalization may be the most critical aspect of burnout in a health care profession like dentistry. Perceiving the patient as an impersonal object rather than a human being might result in detrimental negligence in treatment procedures and disregard of the psychological aspect of treating the patients. Fifth year students had significantly higher scores of depersonalization than 4th year students in both dental schools. No significant differences were found between students of the two schools in the same academic level. These findings might suggest that depersonalization increased with increased patient contact.

Longitudinal studies are necessary to confirm such suggestion, but increasing the student as well as the instructors awareness to such problem is imperative to avoid further development of the problem in the future clinical career of the students.

High scores of personal achievement mean more involvement with the patients, more satisfaction with the profession, and consequently, lower degrees of burnout. The lower scores of personal achievement in 5th year students, revealed in this study, should be viewed as another sign of increasing burnout which was related to increased patient contact in terms of frequency and duration. This scale of burnout is extremely important and should be closely monitored during the clinical training period of dental students and even after their graduation to ensure that the lack of personal achievement has diminished.

### Conclusions

The findings of this study indicated that dental students in the Jordanian universities suffered considerable degrees of burnout as manifested by high degrees of emotional exhaustion and depersonalization.

Longitudinal studies that include pre-clinical dental students as well as young graduates should be carried out to determine how the patterns of burnout vary during the students academic clinical training period and further in their professional lives.

Analytical studies targeting students health and psychology should be carried out regularly to determine the causes and factors related to the high degrees of burnout among dental students.

The curricula of the dental schools in the two universities should be accordingly improved to minimize burnout among the students.

Dental students as well as instructors should be informed about burnout and its elements, to increase their awareness which can alleviate the problem.
